# Dietary supplement users vary in attitudes and sources of dietary supplement information in East and West geographic regions: a cross-sectional study

**DOI:** 10.1186/1472-6882-13-200

**Published:** 2013-07-30

**Authors:** Mary R Rozga, Judith S Stern, Kimber Stanhope, Peter J Havel, Alexandra G Kazaks

**Affiliations:** 1 Department of Nutrition and Exercise Science, Bastyr University, 14500 Juanita Dr. NE, Kenmore, WA 98028, USA; 2 Department of Nutrition, Department of Internal Medicine, University of California, Davis, 1 Shields Avenue, Davis, CA 95616, USA; 3 Department of Molecular Biosciences: SVM, Department of Nutrition, University of California, Davis, 1 Shields Avenue, Davis, CA 95616, USA; 4 Present address: Department of Food Science and Human Nutrition, Michigan State University, 2125 Anthony Hall, East Lansing, MI 48825, USA

**Keywords:** Dietary supplements/utilization, Dietary supplement knowledge, Attitudes, Practices

## Abstract

**Background:**

Over 50% of adults currently use dietary supplements (DS) but manufacturers do not have to prove the safety or efficacy of a DS before it is marketed. Therefore, consumers may be exposed to inaccurate DS information, may lack confidence in choosing appropriate DS and may seek advice for usage. The objective of this study was to examine trends in usage, attitudes, and sources of information regarding DS according to geographic location, demographic group, and lifestyle choices.

**Methods:**

Eligible individuals completed a 10-item researcher-developed survey tool to determine DS use, sources of DS information, and DS-related knowledge and attitudes over the previous year. Healthy participants (637 individuals aged 21–75 years) from two population-based cohorts that had been recruited for lipoprotein assessment studies at Tufts University in Boston, Massachusetts and University of California at Davis. Outcome measures included participants’ use, beliefs regarding essentiality of DS, confidence in choosing appropriate DS, and sources of information on DS. Univariate and multivariate logistic regression were utilized to examine differences in survey responses between groups.

**Results:**

Of the total population 72.7% reported taking dietary supplements in the previous year. Those living on the West Coast (80.3%) had greater use than those living on the East Coast (60.7%). Those on the East Coast were more likely to believe DS were essential to health (48.7%) and to feel confident in choosing DS that were appropriate for them (51.0%). Overall, physicians were the most frequent source of DS information for more than 50% of participants on both coasts.

**Conclusion:**

Because DS usage is widespread, health care providers and nutrition educators must encourage patients to discuss their DS use and be equipped to provide information conducive to safe, efficacious consumption. Tailoring interventions for healthcare providers, media sources, industry, and the public may allow for dissemination of up-to-date information regarding DS.

## Background

Rates of dietary supplement (DS) usage have increased over the past decades and the National Center for Health Statistics reports over 50% of adults used DS from 2003–2006 [1]. In the present study, DS encompass vitamins, minerals and herbs as well as other products such as coenzyme Q10, fish oil, and alpha lipoic acid. Adults may use DS to self-manage specific ailments or for general health reasons, as was found in three surveys of older adults, cancer survivors, and adults over age 18 using supplements [2-4]. Though DS are regulated by the FDA, manufacturers do not have to prove the safety or efficacy of a DS before it is produced, sold, or marketed [5]. Consequently, there are many untested products in the marketplace. It is crucial that consumers are adequately informed about DS from reputable sources and to understand how these products may interact with other DS, food, or medications [6].

It is uncertain whether DS usage is rising due to an increased need for self-treatment due to rising medical costs [7], or if manufacturers are marketing their products more effectively. Few studies have investigated consumer motivations at the root of supplement usage. In a 2001 report, researchers found that many women believed supplement use would serve as “insurance” against illness [8]. While this finding implies that consumers may believe supplementation to be essential to health, the current study asked healthy consumers this question directly. More importantly, since there are no regulations for dispensing evidence-based supplement information, consumers may not feel confident in choosing DS that are appropriate. In this study, the authors examined whether study participants believed DS were essential for health and how confident subjects felt in choosing DS.

An additional concern is the variety of resources of DS information available to consumers that may or may not be accurate. A 2007 survey of Americans 60 years and older revealed that 73% of participants reported television as the most common source of information, followed distantly by magazines and radio, newspapers, friends and store displays [2]. This study was in agreement with a study of women from an urban health clinic in which the authors concluded the most common sources of DS information to be radio and television, followed by family, newspapers and magazines, and friends. The least common sources cited were physicians, the internet, health food stores and registered nurses [9]. Likewise, a study of adults 18 years and older revealed that family, friends, and written materials were the leading sources of information, and health care professionals were the least frequently cited source of information [4]. Finally, a study of consumers in an urban health food store found that 41% of subjects relied on retail staff for DS information, followed by the media, alternative medicine care providers and doctors, nurses and pharmacists [10]. Though many studies have attempted to describe sources of DS information, they are often limited to very specific or disease-related populations, and information is lacking for the general, healthy population.

This objective of this study was to examine two separate geographic regions of the United States to test for overall trends and congruence in the percentage of participants who use DS and to examine the perceived essentiality of DS use to health, the reported confidence in choosing appropriate DS, and sources that inform supplement usage. The data may be used to develop accurate, appropriate information for dissemination to target groups through the most effective media.

## Methods

### Participant recruitment

This was a cross-sectional study in which data was collected from two population-based cohorts recruited for lipoprotein assessment studies at Tufts University in Boston, Massachusetts and University of California at Davis (UCD) between June 2008 and August 2009. Study protocols had institutional review board approval from both institutions and informed consent was obtained from each participant. Participants were recruited by advertisements, press releases, and notices. The study sample included 653 healthy volunteers aged 21–75 (49.5% males), that were matched for age and sex by geographic location. Inclusion criteria included HDL-C levels ≥40 mg/dL, LDL-C <160 mg/dL, TG levels < 200 mg/dL, and fasting glucose < 126 mg/dL. Exclusion criteria included use of drugs affecting lipoprotein metabolism, use of drugs for diabetes treatment, use of illegal drugs, hormone replacement therapy, current/recent CHD/CVD, current/recent diabetes, current/recent hepatic diseases, current/recent renal diseases, current/recent cancer, and current/recent hospitalizations (excluding infectious diseases and external injuries). To ensure eligibility, a medical history, blood pressure, heart rate, anthropometric data and blood samples from participants were collected and evaluated.

### DS assessment

Eligible individuals completed a researcher-developed survey tool to determine dietary supplement use, knowledge and attitudes over the past year. Full label information about each supplement was requested. Other questionnaire items focused on whether DS are essential for health, whether specific supplements had been recommended by a doctor, confidence in understanding which supplements were right for the individual, and where the individual got information about the DS (Additional file 1).

### Participant variables

#### Geographic location

Categorization by geographic location refers to the study location; the Tufts University subjects were considered “East Coast” and the UCD subjects were considered “West Coast”.

#### Sex

Subjects were placed into a “male” or “female” category. If subjects did not answer this question, they were excluded from analyses regarding sex.

#### Age

Subjects were divided into three age groups: 21–39, 40–59, and 60–75. If the subjects did not answer this question, they were excluded from analyses regarding age.

#### BMI

Body Mass Index (BMI) was calculated from self-reported height and weight and presented in units of kg/m^2^. A BMI between 18.5 and 24.9 indicates a normal weight status, a BMI between 25.0 and 29.9 was classified as overweight, and a BMI over 30.0 was defined as obese. Only 15 participants (2.3%) had a BMI <18.5, and these participants were eliminated from analysis. Participants over three standard deviations above the mean were considered outliers and eliminated from analysis regarding BMI (n = 10).

#### Physical activity, alcohol use, and tobacco use

Participants self-reported whether they engaged in regular weekly physical activity, alcohol consumption, and tobacco consumption.

### Statistical analysis

A chi-squared test for independence was used to detect relationships between group inclusion and geographic location and to compare sources of information; results for categorical variables are presented as n(%). Student’s t-tests were used to compare continuous variables between geographic locations and results are presented as Mean ± SD. Univariate and multivariate logistic regression was conducted to determine the odds of using supplements, believing supplements were essential to health, and feeling confident choosing appropriate supplements by demographic group and lifestyle factors. Odds ratios for believing supplements were essential to health compare those who responded “Yes, Essential” to those who responded either “No, Not Essential” or “Don’t Know”. Odds ratios for feeling confident choosing appropriate supplements compare those who responded “Agree” to those who responded either “Neutral”, “Disagree”, or “Don’t Know”. Multivariate logistic regression controlled for participant age, sex, BMI, physical activity, tobacco and alcohol use in addition to the primary independent variable, geographic location. Stata 12.0 was used to conduct statistical analyses. An alpha level of 0.05 was set to denote statistical significance.

## Results

### East vs. West Coast participants

Of the participants in this study, 72.7% used DS in the past year. Those on the West Coast were more likely to be supplement users than those on the East Coast (p < 0.001), and those living on the East Coast had a significantly higher BMI (p < 0.001), were less likely to engage in physical activity (p = 0.046), were less likely to consume alcohol (p < 0.001), and were more likely to consume tobacco (p < 0.001) compared with those living on the West Coast. The populations did not significantly vary by age, or sex (Table 1).

**Table 1:** Demographics and supplement use for all participants and divided into East Coast and West Coast groups

	**Total**	**East coast**	**West coast**	**p-value**^**a**^
Supplement use (n = 637)^b^				
Yes	463 (72.7)	150 (60.7)	313 (80.3)	<0.001*
No	174 (27.3)	97 (39.3)	77 (19.7)	
Age (n = 631)^bc^	47.2 ± 14.8	47.5 ± 12.8	47.0 ± 15.9	0.69
Sex (n = 629)^bd^				
Male	310 (49.3)	123 (51.5)	187 (48.0)	0.39
Female	319 (50.7)	116 (48.5)	203 (52.1)	
BMI (kg/m^2^) (n = 606)^b^	26.5 ± 4.8	28.2 ± 5.1	25.5 ± 4.3	<0.001*
Physical activity (n = 620)^b^				
Yes	502 (81.0)	180 (76.9)	322 (83.4)	0.046*
No	118 (19.0)	54 (23.1)	64 (16.6)	
Alcohol use (n = 632)^b^				
Yes	411 (65.0)	121 (49.8)	290 (74.6)	<0.001*
No	221 (35.0)	122 (50.2)	99 (25.5)	
Tobacco use (n = 631)^b^				
Yes	80 (12.7)	67 (27.7)	13 (3.3)	<0.001*
No	551 (87.3)	175 (72.3)	376 (96.7)	

^a^Compares East Coast to West Coast Participants.

^**b**^n may vary between variables due to missing or incomplete data.

^**c**^Continuous variables expressed in Mean ± SD.

^d^Categorical variables expressed as n (%).

*Denotes statistical significance p < 0.05.

### Supplement usage

Multivariate logistic regression revealed that even after adjustment for demographic and lifestyle factors, those on the West Coast were still significantly more likely to consume DS (adjusted OR: 1.88 (1.20, 2.93); p = 0.005). The oldest age group (60–75 years) had the highest proportion of participants who used any supplement (84.1%) compared with 40–59 year and 21–39 year groups and were significantly more likely to consume DS compared to the youngest age group (adjusted OR: 2.04 (1.12, 3.70); p = 0.02)*.* Compared to males, females were significantly more likely to consume any DS (63.8% vs. 82.8% respectively) (adjusted OR: 2.48 (1.64, 3.74); p < 0.001) (Table 2).

**Table 2:** Supplement use by participant characteristics

	**Any supplement use**	**None**	**Odds ratio, raw (95% CI)**	**Odds ratio, adjusted (95% CI)**^**a**^
Location (n = 637)^bc^				
East Coast	150 (60.7)	97 (39.3)	Ref	Ref
West Coast	313 (80.3)	77 (19.7)	2.62 (1.83, 3.75)***	1.88 (1.20, 2.93)**
Age (n = 628)				
21–39	125 (66.1)	64 (33.9)	*Ref*	*Ref*
40–59	216 (71.8)	85 (28.2)	*1.30 (0.9, 1.9)*	*1.37 (0.87, 2.16)*
60-75	116 (84.1)	22 (15.9)	2.70 (1.56, 4.66)***	2.04 (1.12,3.70)*
Sex (n = 629)				
Male	198 (63.9)	112 (36.1)	Ref	Ref
Female	264 (82.8)	55 (17.2)	2.72 (1.87, 3.94)***	2.48 (1.64, 3.74)***
BMI (n = 637)				
Normal weight	218 (82.0)	48 (18.1)	Ref	Ref
Overweight	134 (65.4)	71 (34.6)	0.42 (0.27, 0.64)***	0.48 (0.30, 0.76)**
Obese	111 (66.9)	55 (33.1)	0.44 (0.28, 0.70)***	0.58 (0.35, 0.97)*
Physical activity (n = 620)				
Yes	380 (75.7)	122 (24.3)	2.00 (1.30, 3.04)**	1.87 (1.18, 2.99)**
No	72 (61.0)	46 (39.0)	Ref	Ref
Alcohol use (n = 632)				
Yes	314 (76.4)	97 (23.6)	1.60 (1.11, 2.29)*	1.31 (0.85, 2.00)
No	148 (67.0)	73 (33.0)	Ref	Ref
Tobacco use (n = 631)				
Yes	43 (53.8)	37 (46.3)	0.37 (0.23, 0.60)***	0.48 (0.28, 0.84)*
No	418 (75.9)	133 (24.1)	Ref	Ref

^a^Adjusted odds ratios controlled for all independent variables.

^b^Data expressed as n(%).

^c^n may vary between variables due to missing or incomplete data.

*p < 0.05; **p < 0.01; ***p < 0.001.

Those in the “normal weight” category were more likely to consume any DS, compared with those in the “overweight” and “obese” BMI groups (adjusted OR: 0.48 (0.30, 0.76); p = 0.002 and 0.58 (0.35, 0.97); p = 0.04 respectively). Those who engaged in regular physical activity were more likely to consume a supplement compared with those who did not engage in physical activity (adjusted OR: 1.87 (1.18, 2.99); p = 0.008) and those who were tobacco users were less likely to use DS (adjusted OR: 0.48 (0.28, 0.84); p = 0.01) (Table 2).

### Attitudes about DS supplementation

In this study, 41.7% of all participants believed DS to be essential to health, however only 44.4% felt confident in choosing appropriate DS for themselves (data not shown). Responses to the question “Do you consider taking vitamins or other supplements such as minerals and herbs to be essential for health?” were significantly different between participants according to geographic location (adjusted OR for believing DS to be essential: 0.45 (0.29, 0.72) for West compared to East Coast participants; p = 0.001). Results varied for other demographic and lifestyle groups, but were attenuated after adjustment, and only supplement use remained a positive predictor of belief in essentiality (adjusted OR for believing DS to be essential: 11.37 (6.35, 20.39); p < 0.001) (Table 3).

**Table 3:** Participant responses to belief that supplements are essential for health by demographic groups

	**Yes, essential**	**No, not essential**	**Don’t know**	**Odds ratio, raw (95% CI)**^**a**^	**Odds ratio, adjusted (95% CI)**^**b**^
Geographic location (n = 618)^cd^					
East	113 (48.7)	78 (33.6)	41 (17.7)	Ref	Ref
West	15 (30.7)	187 (48.5)	42 (10.9)	0.72 (0.52, 1.00)	0.45 (0.29, 0.72)**
Sex (n = 610)					
Male	111 (36.8)	149 (49.3)	42 (13.9)	Ref	Ref
Female	158 (51.3)	112 (36.4)	38 (12.3)	1.81 (1.31, 2.50)***	1.31 (0.89, 1.91)
Age group (n = 609)^d^					
21–39	63 (34.2)	102 (55.4)	19 (10.3)	Ref	Ref
40–59	130 (45.0)	113 (39.1)	46 (15.9)	1.57 (1.07, 2.30)*	1.18 (0.76, 1.84)
60-75	73 (53.6)	47 (34.6)	16 (11.8)	2.22 (1.41, 3.51)**	1.65 (0.98, 2.77)
BMI (n = 618)					
Normal weight	119 (46.0)	111 (42.9)	29 (11.2)	Ref	Ref
Overweight	73 (36.7)	95 (47.7)	31 (15.6)	0.68 (0.47, 0.99)*	0.82 (0.52, 1.28)
Obese	78 (48.8)	59 (36.9)	23 (14.4)	1.11 (0.75, 1.66)	1.27 (0.79, 2.05)
Supplement use (n = 618)					
No	20 (12.1)	107 (64.5)	39 (23.5)	Ref	Ref
Yes	250 (55.3)	158 (35.0)	44 (9.7)	9.03 (5.46, 14.9)*	11.37 (6.35, 20.39)***
Physical activity (n = 603)					
Yes	225 (46.0)	200 (40.9)	64 (13.1)	1.70 (1.11, 2.61)*	1.55 (0.94, 2.55)
No	38 (33.3)	60 (52.6)	16 (14.0)	Ref	Ref
Alcohol use (n = 613)					
Yes	162 (40.8)	185 (46.6)	50 (12.6)	0.70 (0.50, 0.98)*	0.70 (0.47, 1.05)
No	107 (49.5)	77 (35.7)	32 (14.8)	Ref	Ref
Tobacco use (n = 612)					
Yes	34 (44.7)	26 (34.2)	16 (21.1)	1.03 (0.64, 1.68)	1.30 (0.68, 2.49)
No	235 (43.8)	235 (43.8)	66 (12.3)	Ref	Ref

^a^Odds Ratio is the odds of believing supplements are essential compared to other responses.

^b^Adjusted odds ratios are controlled for all independent variables.

^c^n may vary between variables due to missing or incomplete data.

^d^Data expressed as n(%).

*p < 0.05; **p < 0.01; ***p < 0.001.

Those living in the West were less likely to agree that they were confidant in understanding which vitamins, minerals, botanicals and other supplements were right for them compared with those in the West (adjusted OR for feeling confident: 0.60 (0.40,0.89); p = 0.01. No significant differences in participant reports of confidence were noted according to sex, age, BMI, supplement usage, physical activity, or alcohol or tobacco use (Table 4).

**Table 4:** Participant responses to confidence in choosing appropriate supplementation by demographic groups

	**Agree**	**Neutral**	**Disagree**	**Don’t know**	**Odds ratio, raw (95% CI)**^**a**^	**Odds ratio, adjusted (95% CI)**^**b**^
Geographic location (n = 631)^cd^						
East	123 (51.0)	58 (24.1)	17 (7.1)	43 (17.8)	Ref	Ref
West	165 (42.3)	112 (28.7)	70 (18.0)	43 (11.0)	0.70 (0.51, 0.97)*	0.60 (0.40, 0.89)*
Sex (n = 623)						
Male	136 (44.4)	87 (28.4)	42 (13.7)	41 (13.4)	Ref	Ref
Female	149 (47.0)	81 (25.6)	45 (14.2)	42 (13.3)	1.10 (0.81, 1.52)	0.98 (0.69, 1.38)
Age group (n = 593)						
21–39	84 (44.9)	64 (34.2)	20 (10.7)	19 (10.2)	Ref	Ref
40–59	131 (44.1)	73 (24.6)	45 (15.2)	48 (16.2)	0.97 (0.67, 1.40)	0.86 (0.58, 1.27)
60-75	70 (50.7)	28 (20.3)	22 (15.9)	18 (13.0)	1.26 (0.81, 1.96)	1.17 (0.73, 1.86)
BMI (n = 600)						
Normal weight	123 (46.6)	78 (29.6)	28 (10.6)	35 (13.3)	Ref	Ref
Overweight	85 (41.9)	58 (28.6)	32 (15.8)	28 (13.8)	0.83 (0.57, 1.19)	0.78 (0.53, 1.16)
Obese	80 (48.8)	34 (20.7)	27 (16.5)	23 (14.0)	1.09 (0.74, 1.61)	0.97 (0.63, 1.48)
Supplement use (n = 631)						
No	68 (48.00)	47 (27.7)	19 (11.2)	36 (21.2)	Ref	Ref
Yes	220 (47.7)	123 (26.7)	68 (14.8)	50 (10.9)	0.84 (0.72, 0.98)*	1.32 (0.88, 1.98)
Physical activity (n = 625)						
Yes	232 (46.7)	135 (27.2)	68 (13.7)	62 (12.5)	1.30 (0.87, 1.96)	1.23 (0.80, 1.89)
No	47 (40.2)	33 (28.2)	16 (13.7)	21 (18.0)	Ref	Ref
Alcohol use (n = 638)						
Yes	181 (44.3)	114 (27.9)	65 (15.9)	49 (12.0)	0.86 (0.62, 1.19)	0.95 (0.66, 1.36)
No	104 (47.9)	54 (24.9)	22 (10.1)	37 (17.1)	Ref	Ref
Tobacco use (n = 637)						
Yes	34 (43.6	21 (26.9)	147 (26.9)	17 (21.8)	0.91 (0.56, 1.47)	0.77 (0.45, 1.33)
No	251 (45.9)	147 (26.9)	80 (14.6)	69 (12.6)	Ref	Ref

^a^Odds Ratio is the odds of feeling confident choosing appropriate supplementation compared to other responses.

^b^Adjusted odds ratios are controlled for all independent variables.

^c^n may vary between variables due to missing or incomplete data.

^d^Data expressed as n(%).

*p < 0.05; **p < 0.01; ***p < 0.001.

### Sources of DS information

The most common source of information subjects referred to for DS information was physicians followed by books, magazines/journals/the news, the internet, DS labels, family, friends, and health food retailers.

**Figure 1:**
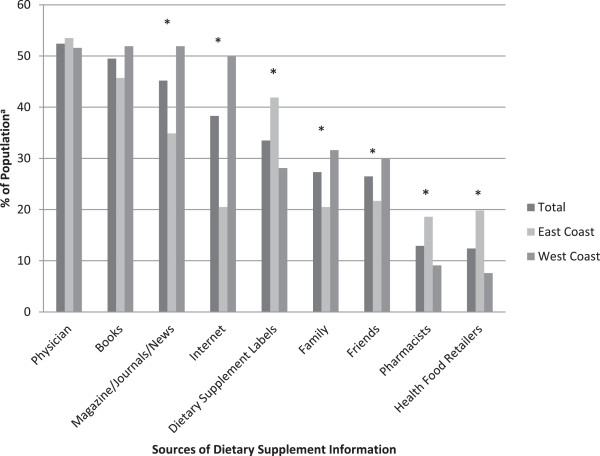
**Sources for East and West Coast participant dietary supplement information. **^a^Subjects were able to choose as many sources as applied. Cumulative values may exceed 100%. * p < 0.05 for differences between East Coast and West Coast participants.

There were marked differences in sources of DS information between geographic locations. Considered together, both groups considered physicians to be the primary sources of information (52.4%), and there was no significant difference between East and West Coast respondents (p = 0.645) (Figure 1). Among all participants, 30.4% described that their doctors recommended specific DS, though there were no significant differences in the proportions of participants recommended DS according to geographic location, BMI, physical activity, or tobacco or alcohol use. Participants were more likely to have received a doctor recommendation for DS if they were female (44.4% vs. 17.0% for males; p < 0.001) or in the oldest age group (45.7% for 60–75 years compared with 31.8% for 40–59 years and 17.6% for 21–39 years; p < 0.001) (data not shown). Apart from physicians and books, other sources of information varied between groups: Those on the East Coast relied more heavily on DS labels, while those on the West Coast relied more heavily on magazines/journals/news and the internet (Figure 1).

## Discussion

### DS usage

A majority of subjects in this study were supplement users, in agreement with the National Center for Health Statistics [1], but in contrast to NHANES data in which only 49% of the population were DS users and the 2007 National Health Interview Survey (NHIS) in which the authors describe only 17.9% of healthy adults in the United States consumed DS [11]. These differences may be attributed both to the volunteer nature of the participants and the geographic locations in which the participants reside in this study. Females and older participants were more likely to be DS users compared with males and younger participants, which is consistent with 2003–2006 NHANES data [12] and 2007 NHIS data [11], though the current data was collected more recently from 2008–2009. To our knowledge, there are no prior studies reporting DS use in healthy individuals by geographic location. In this analysis, participants living on the West Coast had a higher likelihood of DS use compared with those living on the East Coast. Interestingly, despite higher usage, West Coast participants were less likely than East Coast participants to believe that DS were essential for health and were less likely to feel confident choosing appropriate DS, though the later results were not significant after adjustment for demographic and lifestyle factors. Thus, future studies are needed to examine reasons for varying usage between these populations. Similarly to NHANES data [12], this study demonstrates that participants classified as obese had lower DS use compared with those with a normal weight status. However, in the current data those in the overweight group had usage patterns more similar to the obese group while in the NHANES data, they were more similar to those in the normal weight group.

### Attitudes regarding DS supplementation

As medical costs and information about personal healthcare increase, people are turning to alternative therapies and self-treatment, and supplement usage has become mainstream [1]. The prevalence of perceived essentiality of DS and the lack of confidence in choosing DS highlight the opportunity for this topic to be addressed in the traditional medical care setting. Previous research has suggested that patients may be hesitant to initiate conversations about DS with their physicians, creating the possibility for inappropriate dosing and drug interactions [6,13]. This hesitancy to discuss DS use with health care providers is demonstrated in the 2007 NHIS, in which the authors found that only 43.5% of the study population of healthy adults in the United States disclosed DS use to their medical doctors [11], and in a 2008 study conducted by Mehta, et al. which revealed that only about one third of DS users reported use to their conventional health care providers [14]. The need for individualized DS education is demonstrated by the fact that less than 50% of participants were confident in choosing a correct supplement for themselves, despite over 70% of subjects being DS users. To meet the needs of individuals who use DS health care providers and nutrition educators must be prepared to address trends in DS usage and stay current on information about specific DS for diverse patient groups.

### Sources of DS information

This study found that physicians were the most common source for DS information among male and female subjects ages 21–75 at two geographic locations. This finding is similar to those found in a cohort of cancer survivors [3], but are in disagreement with other studies examining healthy participants: A 2004 study of women participants describes health care providers as among the least common sources of information [9], and over half of a population of randomly selected adults from a military family practice did not share their alternative health care practices with their physicians [15]. Archer et al. found that the most common source of information was health food retailers, which was the least common source in this study, and physicians were one of the lowest sources [10], though the population in this study was drawn from a natural food store. Physicians were also among the lowest sources in a study of subjects from Minneapolis/St. Paul and in a study of WIC adult participants from Kansas and Wisconsin [4,16]. A possible reason for this discrepancy may be that the questionnaire in this study did not include TV and radio, which have been noted as primary sources of DS information, as a discrete category and instead included “the news” with journal articles and magazines. Still, this difference in classification does not account for the “physicians” becoming a primary source of information from being one of the least referred to sources, and this finding may be reflective of the geographic locations studied or of secular changes in the population in general. Though the results of this study do not agree with many other studies, they are encouraging, as physicians may be more reputable sources of scientific information compared with the media or family and friends. The findings of this study support a need for training in DS among health care providers, as patients expect physicians to have the best information on DS to optimize health. In a 2003 study, authors tested knowledge of clinically relevant DS and found that, on average, physicians correctly answered only 46% of the twenty questions asked [17]. A later revised survey (2006) revealed that DS knowledge among 374 physicians and physician assistants (PAs) had increased to 70.3%. In this same study, an additional survey was conducted to understand how confident physicians felt in providing DS information and authors discovered physicians and PAs were 55.5% confident in this task [18]. In a recent study of DS knowledge among military physicians, 65% of physicians did not feel they had a reliable source of DS information [19]. The results of the current study suggest that provider conversations about patient-directed DS use are increasing. Improved access to DS knowledge and effective provider-patient communication may lead to more efficacious usage and fewer possible adverse interactions with other supplements, diet, and medications.

### Strengths and limitations

To our knowledge, this is one of the first studies to examine attitudes concerning DS use in a healthy population and the first study to examine attitudes and sources of information regarding DS by geographic location in sex-and age-matched populations. A limitation of this study was the volunteer nature of the study population; individuals agreeing to participate may not be representative of the general population in terms DS attitudes and usage, and findings may not be generalizable to the population of healthy adults in the United States. The data presented here demonstrates that those living on the East and West Coasts may have higher rates of DS usage compared with the general population of healthy adults living in the United States; thus, results from this study should be applied to individuals living in these locations only.

This is a cross-sectional study and, therefore, gives only a snapshot of participants’ attitudes and sources of DS information at the time of the survey. Since that time the internet has become a significant and substantial source of health information. It is possible that current assessments would indicate that online information has a more important role. Also, participants generally did not list any additional information sources such as billboards, television commercials, or radio, missing: that were not included in the main questionnaire items.

## Conclusion

Nearly 73% of this population of healthy subjects reported using DS in the prior year, with females, older participants, and those living on the West Coast tending toward higher supplementation use. Over 40% of participants believed DS to be essential to health, however only 44.4% felt confident in choosing appropriate DS for themselves. There were differences in these attitudes according to geographic location, sex, age, physical activity, and alcohol use. The most common source of DS information was physicians; who may be perceived as providers of more accurate information than other sources. Health care providers and nutrition educators must encourage patients to discuss their DS use and be equipped to provide information conducive to safe, effective, and practical consumption.

## Abbreviations

DS: Dietary supplements; NHANES: National health and nutrition examination survey; PA: Physician’s assistant; BMI: Body mass index; NHIS: National health interview survey.

## Competing interests

The authors declare that they have no competing interests.

## Authors’ contributions

MR participated in the design of the current study, performed the statistical analysis, and drafted the manuscript. JS and AK participated in the study design for collection of supplement data as part of the lipoprotein study, in the design of the current study, and in drafting and editing the manuscript. PH was the Principle Investigator of the original study, and KS was the Study Supervisor. All authors read and approved the final manuscript.

## Pre-publication history

The pre-publication history for this paper can be accessed here:

http://www.biomedcentral.com/1472-6882/13/200/prepub

## Supplementary Material

Additional file 1 **Dietary Supplement Questionnaire.** Questionnaire used to assess participant attitudes about DS as well as sources of DS information. Click here for file
