# Supplementary material for: Dietary supplement users vary in attitudes and sources of dietary supplement information in East and West geographic regions: a cross-sectional study
Source: BMC Complement Altern Med. 2013 Jul 30;13:200. doi: 10.1186/1472-6882-13-200 (PMC3737081; doi:10.1186/1472-6882-13-200)
Supplement: Additional file 1 — Dietary Supplement Questionnaire. Questionnaire used to assess participant attitudes about DS as well as sources of DS information. [file 1472-6882-13-200-S1.docx]

**DIETARY SUPPLEMENT QUESTIONNAIRE**

**Instructions:**

A. You can fill this out at home and bring it with you to your visit.

B. Or, bring all your supplements with you to the visit and fill out the forms at that time.

**Please put an X by the answer you choose for each question.**

Even if you have not used dietary supplements, please answer as many questions as you can.

Please add comments if you want to tell us more about your answer.

| **1. Have you used or taken any vitamins, minerals, herbal products or other dietary supplements in the past year?** | **_____YES**  **_____NO**  **_____NOT SURE** |
| --- | --- |

If you are not sure what dietary supplements are, here are a few examples:

**Some Examples of Dietary Supplements**

|  | **Vitamins** |  | **Herbs or botanicals** |  | **Minerals** |  | **Other supplements** |
| --- | --- | --- | --- | --- | --- | --- | --- |
|  | Multivitamin |  | Echinacea |  | Calcium |  | Coenzyme Q10 |
|  | Vitamin E |  | Garlic |  | Chromium |  | Fish oil |
|  | Vitamin C |  | Ginkgo |  | Iron |  | Glucosamine |
|  | Vitamin A |  | Ginseng |  | Magnesium |  | Melatonin |
|  | Beta Carotene |  | Kava |  | Potassium |  | Omega-3’s |
|  | B vitamins |  | St John’s wort |  | Selenium |  | Alpha Lipoic Acid |
|  | Folic acid |  | Grape Seed Extract |  | Zinc |  | Acetyl-L-Carnitine |
|  | Niacin |  | Saw palmetto |  | Combinations |  |  |
|  | Vitamin D |  |  |  |  |  |  |

**2. Do you consider taking vitamins or other supplements such as minerals and herbs to be essential for your health?**

____YES, ESSENTIAL

____NO, NOT ESSENTIAL

____DON’T KNOW

**Comment:**

**3. Has your doctor recommended that you take specific vitamins, minerals or other supplements for your health?**

____YES

____NO

____DON'T KNOW

If yes, please list which ones:­­­­­­­­­­

For each of these statements, please check whether you agree, feel neutral, disagree or don’t know about the statement

**4. The amount of minerals, vitamins and other substances I get from food is enough for my health needs.**

____ AGREE

____ FEEL NEUTRAL ABOUT

____ DISAGREE

____ DON'T KNOW

**Comment:**

**5. I am confidant that I understand which vitamins, minerals, botanicals and other supplements are right for me.**

____ AGREE

____ FEEL NEUTRAL ABOUT

____ DISAGREE

____ DON'T KNOW

**Comment:**

**6. Labels on dietary supplements help me understand if it is the right supplement for me.**

____ AGREE

____ FEEL NEUTRAL ABOUT

____ DISAGREE

____ DON'T KNOW

**Comment:**

**7. Which of the following sources provide you with information about the dietary supplements that are right for you? (mark as many as you need)**

____BOOKS

____DIETARY SUPPLEMENT LABELS

____FAMILY

____FRIENDS

____HEALTH FOOD RETAILER

____MAGAZINES/JOURNALS/NEWSPAPERS

____MY PHARMACIST

____MY PHYSICIAN

____THE INTERNET

____OTHER SOURCES--Please list:

**Comment:**

**8. Which of the following sources that provide you with information about the dietary supplements do you trust the most? (Please mark no more than 2)**

__­­­­__BOOKS

____DIETARY SUPPLEMENT LABELS

____FAMILY

____FRIENDS

____HEALTH FOOD RETAILER

____MAGAZINES/JOURNALS/NEWSPAPERS

____MY PHARMACIST

____MY PHYSICIAN

____THE INTERNET

____OTHER SOURCES--Please list:

**Comment:**

**9. The main reason I Do or Do Not take supplements is:**

**10.Tell us about the dietary supplements you use**

Complete an information table (found on the next sheets) about the vitamins, minerals, herbal products and other dietary supplements that you regularly take. Use information from the product containers. Use one table per supplement. Fill out as many tables as you need.

- Write full name of the supplement including the brand name and manufacturer. Enter as much information as possible.
- On the days that you took the product, how much did you take on a single day?
- For how long have you been taking this product?
- What is the reason you take this product? Any other comments about the product?

| **Supplement name** |  | | | | | | | |
| --- | --- | --- | --- | --- | --- | --- | --- | --- |
| **Supplement brand/manufacturer** |  | | | | | | | |
| **Form of Product** | Capsule | | Tablet | | Pills | | Soft gels | |
|  | Package/Packet | | Liquid | | Powder | | Wafer | |
|  | Chews | | Granules | | Lozenges | | Gel | |
|  | Other form (specify): | | | | | | | |
| **Amount per day**  **(include how much is in each dose)** |  | | | | | | | |
| **How long taking this product?** | Days | Weeks | | Months | | Years | | Don’t Know |
| **Why I take product**  **and any comments** |  | | | | | | | |
